# Direct Viral Mechanisms Underlying the Onset of HBV-Related Hepatocellular Carcinoma and Implications for Therapeutic Strategies

**DOI:** 10.3390/v18020185

**Published:** 2026-01-29

**Authors:** Simone La Frazia, Alessia Magnapera, Lorenzo Piermatteo, Stefano D’Anna, Leonardo Duca, Valentina Svicher, Romina Salpini

**Affiliations:** 1 Department of Biology, University of Rome Tor Vergata, Via della Ricerca Scientifica, 1, 00133 Rome, Italy; simone.la.frazia@uniroma2.it (S.L.F.); alessiamagnapera97@gmail.com (A.M.); lorenzo.piermatteo@uniroma2.it (L.P.); stefanodanna26@gmail.com (S.D.); 2 Laboratory of Electron Microscopy, National Institute for Infectious Diseases Lazzaro Spallanzani-IRCCS, Viale Portuense 292, 00149 Rome, Italy; duca.leonardo@hotmail.it

**Keywords:** hepatocellular carcinoma, hepatitis B virus, HBV oncoproteins, HBV DNA integration, Nucleos(t)ide analogs, novel anti-HBV drugs, HBV functional cure, HBV sterilizing cure

## Abstract

Hepatocellular carcinoma (HCC) represents the second leading cause of cancer mortality worldwide and is mostly caused by hepatitis B virus (HBV) infection. HBV can induce HCC by an indirect mechanism of continuous necro-inflammation, contributing to hepatocyte damage and promoting cancer, as well as by viral intrinsic factors. Among them, the major contributors to the development of HBV-related HCC are represented by (i) HBV DNA integration in genes modulating cell proliferation, (ii) HBV pro-oncogenic proteins, such as HBx and HBs, and (iii) the accumulation of viral mutations, enhancing the tumorigenic features of HBV proteins. The currently available antiviral treatments, based on the usage of Nucleos(t)ide analogs (NUCs), substantially control HBV replication. However, even a successful NUC treatment does not completely abrogate HCC risk, since it rarely allows achievement of an HBV functional cure, the therapeutic end-point associated with HBsAg loss and more favorable liver outcomes. To date, novel therapeutic strategies based on innovative direct antivirals (nucleic acid polymers, small interfering RNAs, antisense oligonucleotides, covalently closed circular DNA (cccDNA) inhibitors, and capsid assembly modulators) and immune-therapeutics (therapeutic vaccines, checkpoint inhibitors, and Toll-like receptor agonists) are under evaluation in clinical trials. These approaches are showing promising data in terms of an HBV functional cure, thus representing novel strategies that could be beneficial for reducing the burden of HBV-related HCC. Lastly, further efforts in drug development are necessary to identify new compounds that could achieve a sterilizing HBV cure, implying the complete elimination of cccDNA and integrated HBV DNA, the only end-point that completely eradicates HBV and its related oncogenic risk.

## 1. Introduction

Hepatocellular carcinoma (HCC) is the sixth most common cancer and the third cause of cancer mortality worldwide [1], with more than one million deaths predicted in 2040 [2].

Viral hepatitis together with alcohol-associated liver disease and non-alcoholic fatty liver disease (NAFLD) represent the major risk factors for the development of HCC [3,4,5]. Among viral agents, chronic infection with hepatitis B virus (HBV) represents the leading cause of HCC worldwide. In particular, it has been estimated that in 2022, one-third of the 758,725 HCC-related global deaths was attributable to HBV chronic infection [1,6,7,8,9]. Around 80% of all cases of HCC are found in Sub-Saharan Africa and Eastern Asia, which is consistent with the high prevalence of chronic HBV carriers in these regions [2].

Current antiviral treatments, based on the use of Nucleos(t)ide analogs (NUCs), permit substantial control of HBV replication, thus reducing the progression of liver disease toward end-stage liver complications, including HCC. However, the usage of NUCs rarely allows achievement of an HBV functional cure, an ideal therapeutic end-point associated with the loss of HBV surface antigen (HBsAg) and with more favorable liver outcomes. In this light, patients remain at high risk for HCC onset, even under a successful NUC treatment [10].

**Figure 1:**
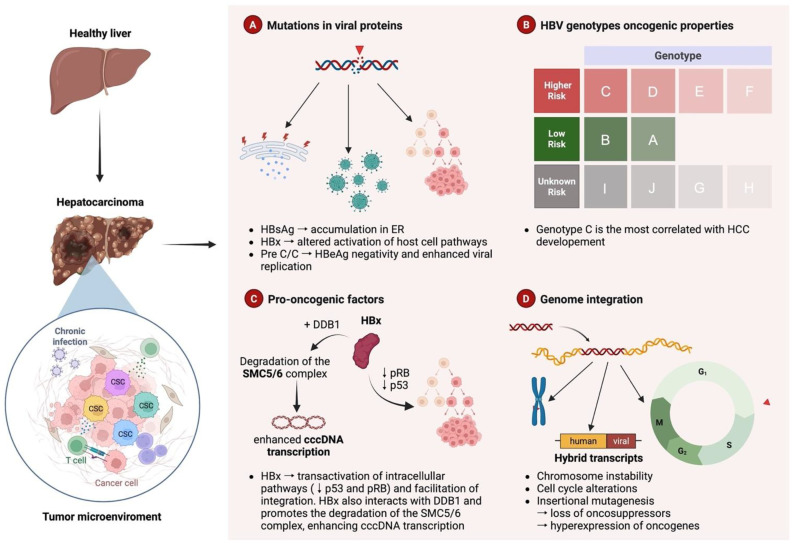
**Main HBV-related mechanisms involved in hepatocarcinogenesis.** Schematic representation of the development from a healthy liver to HCC. This figure summarizes the multifactorial role of HBV in the development of HCC, with the tumor microenvironment being typically characterized by chronic infection, immune evasion, and the presence of cancer cells. The right panel outlines four major intrinsic HBV-related pro-oncogenic mechanisms: (**A**) mutations in viral proteins contributing to liver carcinogenesis. Mutations in HBsAg can cause accumulation of surface antigen in the endoplasmic reticulum (ER), leading to ER stress. Mutations in the HBx result in dysregulated activation of host cellular pathways. Mutations in the precore/core region (PreC/C) lead to HBeAg negativity and enhanced viral replication, which are associated with more aggressive liver disease. (**B**) HBV genotypes and their oncogenic properties: HBV genotype influences the risk of HCC development. Genotype C is most strongly associated with HCC, followed by genotypes D, E, and F (higher risk). Genotypes A and B are associated with a lower risk, while for genotypes G, H, I, and J, there are still limited data on their oncogenic potential. (**C**) Pro-oncogenic factors: HBx exerts pro-oncogenic effects through the interaction with the host factor DDB1, leading to degradation of the SMC5/6 complex and enhancement of covalently closed circular DNA (cccDNA) transcription. HBx also suppresses the tumor suppressor proteins p53 and pRB, facilitating oncogenic signaling and viral genome integration. (**D**) HBV genome integration: HBV DNA integration into the host genome results in chromosomal instability, cell cycle dysregulation, and the formation of human-viral hybrid transcripts. This can cause insertional mutagenesis, leading to loss of tumor suppressor genes and overexpression of oncogenes, ultimately promoting carcinogenesis.

HBV-induced HCC usually develops in an environment of persistent necro-inflammation and continues compensatory hepatocyte regeneration, characterizing HBV chronic infection, thus suggesting an important role of immune-mediated liver damage in the pathogenesis of HCC (Figure 1) [11].

Non-infectious factors exert a substantial synergistic influence on HBV-driven hepatocarcinogenesis by intensifying the chronic inflammatory and fibrogenic milieu established by persistent viral infection [12,13]. Recent clinical evidence from a large cohort of patients with HBV-related cirrhosis receiving first-line antiviral therapy has shown that alcohol consumption is associated with a 20–30% increased risk of liver-related mortality or liver transplantation, with an even higher risk in heavy drinkers, despite effective viral suppression [14].

Metabolic dysfunction, particularly steatosis, insulin resistance, and lipotoxicity activates the JNK, NF-κB, and mTOR pathways, creating a pro-inflammatory and mitogenic environment that amplifies HBV-induced hepatocyte turnover, thus amplifying the oncogenic potential of HBV [15,16,17,18]. Recent multi-omics analyses in HBV-associated HCC have shown upregulation of steroid hormones, primary bile acid, and sphingolipid metabolism, which activate the MAPK/mTOR pathway, establishing a self-amplifying loop that drives lipid metabolic reprogramming and enhances the oncogenic potential of HBV [18]. In untreated chronic hepatitis B (CHB) patients, hepatic steatosis reduces HCC risk (HR 0.45), while additional metabolic dysfunction increases it (HR 1.40) [19]. A recent study demonstrated that, in patients with HBV-HCC undergoing radical resection, 20% had MAFLD; overall, this condition did not affect survival, whereas in the presence of diabetes, it was associated with poorer prognosis [20].

Furthermore, non-modifiable host-related factors, including older age, male sex, sub-Saharan African ethnicity, Asian Pacific Islander ancestry, HLA II, STAT4, IFNL3/4 polymorphisms, and pre-existing cirrhosis are known for their capability to modulate HCC risk, reflecting the multifactorial nature of HBV-associated carcinogenesis [21,22,23,24,25,26,27].

Notably, HBV-related HCC also develops in a relevant number of patients lacking any sign of liver damage (neither inflammation nor cirrhosis) [28], highlighting the existence of intrinsic HBV-mediated pro-oncogenic mechanisms (Figure 1) [29,30,31,32,33,34,35]. In this regard, to date, HBV has been recognized to possess several oncogenic factors, directly contributing to HCC development, regardless of liver inflammation (Figure 1) [35,36].

One of the leading molecular mechanisms that mediates the direct HBV pro-oncogenic properties is represented by the capability of HBV to integrate portions of its viral DNA into the host genome (Figure 1).

Although HBV possesses a reverse transcriptase, integration into the host genome is rare (<1% of infected hepatocytes) and, unlike retroviruses, is not mandatory. However, when it occurs, it may increase the risk of developing HCC [37]. During reverse transcription, double-stranded linear (DSL)-HBV DNA is generated when the viral polymerase fails to complete circularization. This linear form can enter the nucleus and integrate into the host genome via non-homologous end joining (NHEJ). Unlike covalently closed circular DNA (cccDNA), integrated HBV DNA cannot produce viral particles, but it continues to express viral antigens such as HBx and HBsAg, which are known to display pro-oncogenic activities, overall promoting chronic infection, immune evasion, and HCC development.

Moreover, HBV integration can directly involve genes regulating the cell cycle, thus resulting in a perturbation of hepatocyte proliferation and functionality, and it can also induce partial or overall chromosomal instability (deletions, insertions, translocations, and inversions even when far from the location of HBV integration) and, in turn, predispose hepatocytes to neoplastic transformation [38]. Accordingly, the evidence of integrated HBV DNA in cellular genes by cis- and/or trans-mediated mechanisms regulating proliferation in most of HCCs reinforces the direct role of HBV integration in oncogenic transformation (Figure 1) [38,39].

Lastly, HBV integration can lead to the formation of extrachromosomal circular DNA (eDNA), which replicates independently and exhibits genomic instability, facilitating recombination and reintegration into the host genome. As a potent driver of gene amplification, eDNA can increase oncogene copy number and intratumoral heterogeneity and/or function as a mobile transcriptional enhancer, further contributing to liver tumor progression [40].

Among HBV proteins, both the regulatory X protein (HBx) and the surface glycoprotein (HBsAg) are currently recognized as relevant pro-oncogenic factors capable of promoting neoplastic hepatocyte transformation, even in patients with persistent viral suppression under successful antiviral treatment (Figure 1) [37,41].

On the other hand, it is relevant to remark that HBV tends to accumulate several mutations during HBV replication due to two intrinsic characteristics of the virus: (i) its utilization of a viral RNA-dependent DNA polymerase lacking proofreading activity and, thus, prone to inducing mutations, and (ii) its very high viral replication rate with over 10^10^ virions produced per day in active patients. As a result of the high mutational rate and the elevated viral particle production, several HBV genotypes and subgenotypes have evolved over time.

Furthermore, in view of this high degree of genetic heterogeneity characterizing HBV, coupled with the direct pro-oncogenic properties demonstrated for several HBV proteins [42], an increasing number of studies have explored the association of specific mutations occurring in the regions encoding the viral proteins (HBx, HBs, and, to a lesser extent, HBc) with HCC onset. In particular, a significant amount of evidence points to the existence of specific genetic viral signatures contributing to HCC development (Figure 1). In light of this, several studies have proposed how these viral mutations can potentially serve as important prognostic biomarkers of HBV-related disease progression, helping to recognize patients at higher risk for HCC, and, in turn, to facilitate early diagnosis and treatment.

In this review, we will provide a comprehensive overview of the direct mechanisms by which HBV can mediate viral tumorigenesis. Furthermore, we will discuss the current knowledge on the contribution of HBV genetic variability, in terms of both genotypes and specific mutations, in the development of HBV-related HCC. In addition, the currently available drugs and the most innovative pharmacological approaches for the development of therapeutic strategies to prevent or delay the progression of HBV chronic infection and the onset of related HCC will be discussed.

## 2. Mechanisms Underlying the Onset of HBV-Related HCC

The risk of hepatocellular carcinoma (HCC) persists in all HBV-chronically infected patients, but its entity varies significantly across the different clinical phases of HBV infection. In particular, the highest oncogenic risk is generally associated with the immune-active phases such as HBeAg-positive and HBeAg-negative chronic hepatitis B [43]. Indeed, these phases (also referred to as Phase 2 and Phase 4) are characterized by intense necroinflammatory activity (highlighted by elevated ALT levels) and by conspicuous viral replication (HBV DNA > 2000 IU/mL); both of these factors are known to accelerate fibrosis progression and to drive HCC development [44]. It is relevant to remark that during phases 1 and 3 of HBV chronic infection, characterized by low HBV DNA levels and minimal liver inflammation, HCC risk is reduced but not completely abolished, particularly in patients with pre-existing fibrosis or cirrhosis as a consequence of HBV intrinsic oncogenic properties [43]. Furthermore, episodes of viral reactivation, whether spontaneous or therapy-related, enhance oncogenic potential through repeated cycles of hepatocyte damage and cycles of regeneration, which can promote oncogenic transformation. In particular, during HBeAg-positive chronic infection (Phase 1), young individuals (<30 years old) typically exhibit a lower immediate clinical risk; however, the risk of HCC is paradoxically highest in HBeAg-positive patients with moderate baseline viral loads (10^6^–10^8^ IU/mL) than in patients with extremely high levels (>10^8^ IU/mL), as seen in the earliest stages of infection [45,46]. Phase 3 (HBeAg-negative chronic infection) is associated with a lower but persistent risk of HCC, although this risk increases if HBsAg levels remain >1000 IU/mL despite low-level viraemia [43,45,46].

In Phase 5 of HBV infection (Occult HBV Infection or OBI), the risk of developing HCC remains below 1%; nonetheless, this persistent, albeit low, risk highlights the intrinsic pro-oncogenic role of the hepatitis B virus [46].

A strong association between HBV viremia and HCC has been consistently demonstrated, with serum HBV DNA representing one of the most robust predictors of carcinogenesis independent of biochemical or histological activity [47,48]. Indeed, high viral loads can promote HBV DNA integration, upregulate oncogenic viral proteins, and sustain chronic inflammatory signaling, collectively driving malignant transformation [49]. Although long-term antiviral therapy effectively suppresses HBV replication and significantly reduces HCC incidence, residual risk persists, underscoring the central role of viral replication in HBV-related oncogenesis [49].

### 2.1. Oncogenic Properties of the Different HBV Genotypes

To date, based on an intergroup divergence > 8% across the complete genome, HBV has been classified phylogenetically into 9 genotypes, A-I, with a putative 10th genotype H [50,51,52]. Furthermore, >30 different HBV subgenotypes are currently recognized according to an intergroup divergence > 4%.

HBV genotypes display diverse geographical distribution: genotypes A and D are the most predominant in Europe, whereas genotypes B and C are common in Asia; genotype E predominates in Africa, genotype F is frequent in Central America, and genotypes G and H have been found in South America. Beyond their different geographical distribution, HBV genotypes have also been associated with a different risk of liver disease progression and HCC onset. Indeed, several studies have shown that specific mutations characterizing viral genotypes can influence HBV-related disease outcomes, favoring the development of HCC (Figure 1) [53].

Studies involving Asian cohorts have demonstrated that chronic HBV infection caused by genotype C is more likely to cause cirrhosis and HCC than that caused by genotype B (Figure 1) [54,55,56,57]. Similarly, genotype D has been associated with a more severe liver disease than genotype A, and it has been suggested to also play a predictive role in the occurrence of HCC among young patients (Figure 1) [58]. More recently, a study analyzing 100 patients with a long-term follow-up (30 years after the first baseline assessment of HBV infection) observed that HCC developed more frequently in patients with genotype C (33%) than in those with genotypes B (17%), D (2%), and A (0%), respectively, as also confirmed by multivariable analysis (*p* < 0.0001) (Figure 1) [59].

Epidemiological studies have suggested that the African HBV genotype E is associated with the highest risk of developing hepatocellular carcinoma, even in the absence of liver fibrosis/cirrhosis and at a younger age (Figure 1) [60]. Although the mechanisms underlying these pro-oncogenic characteristics have not yet been fully clarified, some specific mutations and deletions in the preS/S and BCP regions have been proposed to play a role in the increased oncogenic potential of genotype E [60].

Despite the relatively limited number of available studies, hepatitis B virus genotype F, particularly subgenotype F1b, has been repeatedly linked to a higher incidence and earlier onset of hepatocellular carcinoma in Latin American and Arctic populations, suggesting a distinct oncogenic potential that warrants further investigation (Figure 1) [61,62]. Limited/no information is available regarding the oncogenic potential of the other HBV genotypes (G, H; I, J) (Figure 1).

### 2.2. The Role of HBx and Its Genetic Variability in HCC Onset

The HBx protein plays a pivotal role in hepatocarcinogenesis by interacting with numerous cellular proteins and activating multiple intracellular signaling pathways, thereby promoting hepatic cell hyperproliferation that contributes to the development of HCC [42,63,64].

HBx is also capable of directly inactivating or indirectly down-regulating various tumor suppressors or senescence-related factors, promoting enhanced hepatocyte survival (Figure 1) [65]. All these HBx-mediated mechanisms are currently recognized as important drivers for the initiation of liver cancers. However, the complexity of different and opposite effects that HBx can mediate in infected cells strongly suggests that this protein can act differentially according to its level of expression and its different composition. This highlights the potential contribution of HBx genetic variability in modulating the role of this oncoprotein in HCC onset.

HBx modulates the cell cycle by interacting with regulators such as cyclins and cyclin-dependent kinases (CDKs), promoting uncontrolled cell proliferation [65]. Additionally, HBx inhibits apoptosis by interfering with apoptotic pathways regulated by proteins such as p53, allowing infected cells to survive longer and accumulate mutations (Figure 1) [64,66].

In particular, HBx is characterized by the presence of two functional domains: the N-terminal domain endowed with anti-apoptotic effect and the C-terminal domain that exhibits pro-apoptotic activity and is involved in transactivation mechanisms. Considering these dual properties, it has been proposed that the capability of HBx to induce HCC may also depend on the equilibrium between the anti-apoptotic N-terminal and the pro-apoptotic C-terminal domains [67,68,69,70].

The protein also plays a role in immune evasion by altering immune signaling pathways, particularly through the activation of nuclear factor-kappa B (NF-κB), enabling the virus to evade immune detection and persist in the host, thereby increasing the risk of HCC [68]. Furthermore, HBx contributes to genomic instability through interactions with DNA repair mechanisms and tumor suppressor genes, leading to mutations and chromosomal abnormalities that drive cancer progression. Finally, HBx influences epigenetic processes, such as DNA methylation and histone modifications, which affect gene expression and facilitate the transformation of normal hepatocytes into cancerous cells (Figure 1) [63,71,72]. HBx facilitates the integration of the HBV genome into the host’s DNA, playing a pivotal role in establishing chronic infection [73]. Some mutations in the HBx gene have been associated with an increased risk of liver carcinogenesis, particularly in individuals with chronic HBV infection. Mutations in the transactivation domain of HBx can lead to altered activation of host cell signaling pathways, such as Activator Protein-1 (AP-1), NF-κB, and Wnt/β-catenin (Figure 1) [74]. Among the mutations detected in HBx, C1653T, C1485T, C1470A, C1479A, and C1575G in patients with chronic hepatitis, the two most frequently associated with HCC are C1485T and C1653T [75]. Specifically, the C1485T mutation enhances the transcriptional activity of the Wnt signaling pathway and activates NF-κB [75]. These alterations promote cell survival, enhance inflammatory responses, and create a microenvironment favorable to tumorigenesis.

Patients with a more severe prognosis due to chronic HBV infection, including HCC, often harbor double mutations at the K130M/V131I positions in the HBx protein, which can be associated with nucleotide alterations in the basal core promoter (BCP) region (A1762T/G1764A), leading to decreased PreC/C RNA synthesis and reduced or absent HBeAg expression [76].

Chiu and colleagues [77] generated transgenic mice expressing either the wild-type (WT) HBx gene from the Asian genotype B HBV or the HBx gene carrying the K130M/V131I mutations. The tumorigenic potential in mice carrying the mutated HBx was significantly greater than in WT HBx mice due to activation of the Akt/FOXO1 (Forkhead Box O-1) signaling pathway and enhanced hepatic inflammation via arachidonic acid metabolism.

A large meta-analysis of 12 and 35 studies investigating the association of I127N/S/T mutations and double K130M and V131I mutations showed that these mutations were statistically significantly associated with the risk of HCC [78,79]. Moreover, in a Chinese prospective study of 2258 HBsAg-positive patients, 61 HCC patients were diagnosed after 36 months of follow-up; of those, 89% had K130M and V131I mutations, confirming that patients carrying these mutations have a higher risk of progressing toward HCC [80].

In vitro studies showed the mutations K130M and V131I increase core RNA levels and the viral replication rate and decrease precore RNA levels, suggesting that these mutations are responsible for the enhancement of RNA replication, pgRNA levels, and core production and for hindering but not abolishing HBeAg secretion [74,78,81,82,83]. Nevertheless, the exact mechanism of HCC development is still unclear, and further functional in vitro and in vivo studies of these mutations are needed.

Notably, deletions of the HBx C terminus are the most frequently reported HBx modifications associated with HCC. A higher rate of truncated HBx has been described in hepatocarcinoma cells rather than in untransformed hepatocytes, highlighting the potential role of these deletions in tumor development [84].

Pu and colleagues [85] demonstrated that mice injected with specific HBx mutant constructs carrying the truncated HBx variant exhibited a significantly higher tumor burden compared to those injected with wild-type (WT) HBx. Histopathological analyses revealed a pronounced inflammatory response associated with high levels of interleukins such as IL-5, IL-6, and IL-1β, a key mediator of tumor invasiveness, progression, and metastasis. Furthermore, the expression of Plasminogen Activator Inhibitor-1 (PAI-1), a proteolytic regulator involved in angiogenesis, was significantly upregulated in these HBx mutant mice.

Recently, HBx has been shown to enhance cccDNA transcription by promoting the degradation of the host antiviral Structural Maintenance of Chromosomes (Smc) 5/6 complex. This occurs via its interaction with the cellular adaptor protein DDB1 (Damage-Specific DNA Binding Protein 1) (Figure 1) [86]. In light of this, targeting this HBx–DDB1 interaction presents a promising therapeutic strategy against HBV.

### 2.3. The Role of Precore/Core Region and Its Genetic Variability in HCC Onset

The precore region of the virus genome plays a crucial role in the replication and morphogenesis of the virus, and it controls the transcriptional initiation for the synthesis of the precore/core mRNA and pregenomic RNA (pgRNA). The PreC/core open reading frame in the HBV genome encodes two viral proteins: HBcAg, constituting HBV capsid, and the accessory HBeAg protein [83]. The core protein is well known to be essential in the regulation of HBV replication, secretion, and pathogenesis, whereas HBeAg has no defined role in HBV replication, but it is widely used as a marker of HBV infectivity [87,88]. These two proteins encompass different antigenic epitopes that are the main targets of CD4- and CD8-T-cell mediated immune responses. Antibodies mediating the immune response to HBeAg and HBcAg are fundamentally crucial in predicting HBV suppression; therefore the absence of antibodies may hinder the efficiency of HBV suppression in infected cells. The mutations in these antigenic regions might occur due to immunological pressure acting on these regions and can alter antigenicity; thus they may drive HBV immune evasion and promote infection persistence. Likewise, mutations might also occur out of these antigenic regions and be related to the progression of liver diseases, suggesting that they may be involved in molecular mechanisms underlying HBV pathogenesis.

Several mutations in the regulatory region of the precore have been associated with an increased progression toward hepatocarcinogenesis [89]. Mutations in the precore region, particularly at nucleotide position 1896 (G1896A), with a guanine (G) to adenine (A) substitution is well known to generate a premature stop codon, preventing the production of HBeAg and resulting in HBeAg negativity. This condition has been reported to affect viral clearance and to exacerbate liver disease progression, including HCC (Figure 1). A meta-analysis, including 18 studies, has also confirmed that the mutation G1896A causes a 2-fold increase in the risk of HCC onset (summary OR = 2.04, 95% CI = 1.41–2.95) [90]. More recently, a study has demonstrated that G1896A promotes tumoral transformation of infected hepatocytes and their enhanced cell survival and growth by activating the extracellular signal-regulated kinase/mitogen-activated protein kinase (ERK/MAPK) signaling pathway [91]. Moreover, according to a metanalysis involving 10 studies, significant correlation with the occurrence of HCC was also found for nucleotide substitution in basal core promoter A1762T (summary OR = 3.96, 95% CI = 1.98–7.92), as well as for G1764A (summary OR = 3.48, 95% CI = 1.99–6.09). Lastly, by analyzing 22 studies, for the double mutation A1762T/G1764A, a 4-fold increase in the risk of HCC was found (summary OR = 3.96, 95% CI = 2.77–5.65), supporting the role of the co-existence of the two mutations in enhancing the pro-oncogenic properties of viral strains [90].

Notably, the precore and core regions are of great interest since several studies reported specific hotspot variations in these regions that are implicated in the risk of HCC. Kim and colleagues [92] reported in a Korean study of 70 patients chronically infected with HBV genotype C that one mutation in the precore region (W28*) and five mutations in the core region (P5H/L/T, E83D, I97F/L, L100I, and Q182K/*) were associated with HCC. Notably, the reported core mutations were mostly localized in regions of MHC I and II-restricted T-cell epitopes, suggesting that immunological pressure driving the occurrence of these mutations may also play a crucial role in HCC progression. Furthermore, only the W28* was strongly correlated with HBeAg negativity after introducing a premature stop codon in the precore region, which blocks HBeAg production. It is well known that HBeAg-negative patients with significant viremia have a higher risk of liver disease progression than HBeAg-positive patients, suggesting that the lack of HBeAg production resulting from the mutation leads to infection persistence, thereby exacerbating liver inflammation and necrosis and overall favoring hepatocarcinoma onset. Accordingly, Malik and colleagues [93] reported in a study of 331 patients infected with HBV genotypes D and A that W28* mutation is present in over half of HCC patients compared to one-fourth of non-HCC patients. However, there is still the need to confirm the data in larger longitudinal and functional studies to better understand its rate of occurrence and to elucidate its specific effect in the oncogenic process.

### 2.4. The Role of the PreS/S Region and Its Genetic Variability in HCC Onset

The S gene contains three different ORFs which code for the L- (Large), M- (Medium) and S- (Small) surface antigens (L-, M-, S- HBsAg), respectively. These three proteins are present in the viral envelope and are involved in the recognition of host cell and virion assembly [94]. They derive from the translation of two different subgenomic RNAs, one of 2.4 kb, which encodes for the L-HBsAg protein, and one of 2.1 kb, which encodes for M- and S- HBsAg proteins. L-HBsAg, encoded by the pre-S1/pre-S2/S gene, is made up of about 389–400 amino acids (according to HBV genotype) and is responsible for viral entry by mediating the binding to the cellular receptor for viral entry. The M-HBsAg is encoded by the pre-S2/S gene and consists of about 281 aa, and its role in viral replication has not yet been fully characterized. Finally, the S-HBsAg, encoded by the S gene, is the most abundant on the virion surface and is also present in circulating subviral [94]. The different HBsAg forms are synthesized in the endoplasmic reticulum where they rapidly undergo dimer and multimer formation via extensive disulphide bonding [95]. This results in budding into the endoplasmic reticulum as either spherical or filamentous empty subviral particles (mainly composed of S-HBsAg), or as virions, a step preceding their final release from the hepatocytes [94]. The accumulation of HBsAg in the endoplasmic reticulum (ER) has been demonstrated to cause ER stress, a condition that can consequently alter several signaling pathways essential for regulating cell proliferation, invasion, cell survival, and apoptosis, posing the basis for the neoplastic transformation of the infected hepatocytes (Figure 1) [37,96,97]. Additionally, the oxidative stress resulting from the abundance of intracellular HBsAg can also directly induce DNA damage, promoting hepatocarcinogenesis [37,98].

Various modifications (such as stop codons/mutations in the S region and deletions in the preS1 and preS2 regions) have been described for their oncogenic potential since they can favor the intrahepatic accumulation of unfolded and misfolded HBsAg in ER. Notably, these unfolded/misfolded proteins can exert cytotoxic effects, and can activate intracellular signaling pathways associated with oxidative stress, thus promoting the transformation of the infected hepatocytes (Figure 1) [99,100].

In particular, deletions in preS1, preS2 are the most common variations found to be associated with HCC in vivo in the literature. In a study examining serum samples from 387 patients from 12 different countries, a correlation analysis showed that deletions in the preS1 region and in the preS2 region were found more frequently in patients with HCC (*p* < 0.05), indicating that they represent a risk factor for the development of hepatocellular carcinoma in clinical studies [101]. In a case–control study involving 160 HBV infected patients with different clinical stages of disease, 37 of them had mutations in Pre-S regions (23%), and 52% of them developed hepatocellular carcinoma [102]. Lastly, in a study including patients in different disease stages, the pre-S deletion rate was 7% in patients with acute HBV infection, in contrast with chronic HBV carriers and HCC patients, which was 37% and 60%, respectively, highlighting that these mutations do not appear immediately but develop with the progression of the disease [103,104]. The correlation of PreS mutants with HCC has also been confirmed in several other studies [105,106,107].

Regarding S-HBsAg, the hydrophobic C-terminal domain (from aa 179 to 226) is the one involved in mediating the transit of surface glycoproteins across the endoplasmic reticulum. Stop codons abrogating the synthesis of HBsAg C terminus and mutations occurring in this domain have been described to cause the production of a stable, glycosylated, but non-secreted chain that can affect HBsAg secretion [108].

In particular, truncated forms of HBsAg have been frequently detected in clinical specimens in HCC patients, with HBV variants containing premature stop codons at several HBsAg positions (such as sW172*, sW182*, and sW196) [109,110,111].

The expression of these truncated S proteins can result in their intracellular accumulation, which can lead to the activation of the endoplasmic reticulum stress pathway, where they can alter regulatory pathways, such as the transactivation of cellular genes including *c-myc*, *c-fos*, and *H-ras oncogenes* and the specific activation of the c-Raf-1/MEK/Erk2 signal transduction cascade, resulting in the induction of enhanced hepatocellular proliferative activity [111]. Similarly, a study has shown that two specific mutations in HBsAg C terminus, P203Q and S210R, alone or in combination, significantly correlated with HCC in vivo [97], hampered HBsAg secretion, and augmented the percentage of cells in the S-phase and in the G2/M-phase in vitro, overall supporting their role in stimulating hepatocytes proliferation, and, in turn, their involvement in HCC development [97].

### 2.5. The Contribution of HBV Integration in HCC Onset

The HBV replication cycle begins when rcDNA is transported to the nucleus and converted into covalently closed circular DNA (cccDNA), which serves as a template for viral RNA, including pregenomic RNA (pgRNA). This pgRNA undergoes reverse transcription to form relaxed circular DNA (rcDNA), which is encapsulated into new virions. A small proportion (10%) of double-stranded linear DNA (dslDNA) is also generated and incorporated into the virions [112]. The dslDNA is replication-defective but capable of integrating into the host genome via DNA break sites non-homologous end joining (NHEJ) [113].

The majority of the research on HBV DNA integration has been focused on its potential to drive HCC. Indeed, the first descriptions of HBV DNA integrated into the host cell genome were from primary HCC tissues and HCC-derived cell lines, prompting suggestions that integrated HBV DNA was causative in tumorigenesis [114]. The reported mechanisms include (1) cis-mediated insertional mutagenesis of HCC-associated genes; (2) induction of chromosomal instability by integrated DNA; and (3) the expression of mutant HBV genes from their persistent integrated form. However, the mechanism of HBV-induced HCC carcinogenesis remains unclear and poorly characterized (Figure 1) [114].

#### 2.5.1. Chromosomal Instability

HBV integration events significantly compromise the integrity of the host genome by inducing chromosomal aberrations, such as structural rearrangements, aneuploidy, and nterchromosomal translocations [37,114,115]. These alterations often result in the inactivation of tumor suppressor genes (e.g., p53) and the amplification of oncogenic drivers (e.g., MYC, TERT) [116,117]. Multiple HBV integration loci are detected in the majority of virus-associated HCCs. Recurrent insertion hotspots involve genes such as Telomerase reverse transcriptase (TERT), lysine methyltransferase 2B (KMT2B), GLI family zinc finger 2 (GLI2), cyclin A2 (CCNA2), cyclin D1 (CCND1), cyclin E1 (CCNE1), and Mixed lineage leukemia-4 (MLL4), which are implicated in malignant transformation and poor clinical outcomes [63,118].

The integration is not confined to late-stage disease or high viremia; in fact, it can occur early during infection and has been observed across the spectrum of liver pathology, from chronic hepatitis and cirrhosis to early and advanced HCC [37]. Svicher and colleagues demonstrated that HBV DNA integration occurs in all HBeAg-negative patients with chronic infections, including those with a limited intrahepatic HBV reservoir, localizing in genes involved in HCC and altering the hepatocyte transcriptome [119], providing confirmation that viral integration is a persistent and irreversible molecular signature with oncogenic potential. Moreover, the integrated HBV DNA, providing a continuous source of viral antigen expression (e.g., HBsAg, HBx), supports chronic immune activation and hepatocyte turnover, further exacerbating genomic damage [120].

Furthermore, recent single-cell and spatial transcriptomic studies have revealed that an integrated HBV genome can be heterogeneously distributed within the tumor microenvironment, contributing to intratumoral diversity and resistance to therapy; in particular, the clonal expansion of hepatocytes harboring integration may promote tumor evolution and treatment escape in the context of immune checkpoint inhibition or kinase inhibitor therapy [121,122].

#### 2.5.2. Insertional Mutagenesis

HBV DNA integration is facilitated through non-homologous end joining (NHEJ), a DNA repair mechanism prone to introducing indels and rearrangements at the host-viral junctions [123]. These integrations often perturb genes regulating cell proliferation and apoptosis. While HBV integration occurs stochastically in non-malignant hepatocytes, clonal expansion of cells harboring integrations near cancer-related loci is a hallmark of HCC [122]. A notable consequence of integration is the formation of HBX-LINE1 fusion transcripts, present in approximately 25% of HCC cases. These chimeric RNAs act as sponges for miR-122, a liver-specific tumor suppressor microRNA, thereby deregulating cell cycle control and enhancing mitotic activity [124]. TERT promoter activation via HBV genome integration leads to telomerase reactivation, a frequent early event in hepatocarcinogenesis [125].

#### 2.5.3. Expression of Mutated Viral Proteins

Integrated HBV DNA retains the capacity to express viral proteins through host transcriptional machinery and alternative polyadenylation. Notably, hepatitis B surface antigen (HBsAg) and structurally altered forms of the HBx protein, including C-terminal truncations and viral-host chimeras, can be synthesized from integrated templates. Truncated HBx variants have been associated with poor clinical outcomes and are hypothesized to drive oncogenesis through downregulation of metabolic tumor suppressors such as TXNIP [63]. Both covalently closed circular DNA (cccDNA) and integrated HBV sequences may persist long-term within hepatocytes. Collectively, the oncogenic potential of HBV stems from the interplay of direct mechanisms, such as viral protein expression, and indirect mechanisms, including chronic inflammation, immune-mediated hepatocyte turnover, and genome-destabilizing integration events.

### 2.6. The Role of HDV Coinfection in the Development of HCC

To date, epidemiological data indicate that both simultaneous infection with HBV-HDV (coinfection) and infection with HDV in individuals with pre-existing chronic HBV (superinfection) result in more severe hepatitis than HBV monoinfection alone [126,127]. Chronic HDV infection is recognized as a major risk factor for HCC, increasing the risk by two- to threefold compared to HBV monoinfection. It leads to the development of cirrhosis in approximately 15% of cases within the first 2 years and in up to 60% of cases within 5 to 10 years [128].

The enhancement of oncogenic potential associated with HBV-HDV coinfection is determined by multiple mechanisms. HDV is a cytopathic virus and causes direct damage to hepatocytes, promoting persistent inflammation and fibrogenesis [129]. This pro-fibrotic hepatic microenvironment supports tumorigenesis through the activation of hepatic stellate cells, the induction of pro-inflammatory cytokine cascades, and the production of oxidative stress leading to DNA damage [130].

Furthermore, HDV infection induces strong and chronic immune responses responsible for immune-mediated hepatocyte destruction [131,132]. This inflammatory environment contributes to genomic instability, selective survival, and clonal expansion of hepatocytes with advantageous mutations and increased pressure for HBV DNA integration into the host genome [133]. This aspect may be further exacerbated by the degree of viral genetic variability, which can promote HDV evasion of immune responses and has led to viral differentiation into genotypes and subgenotypes with potentially different pathobiological properties [128,134,135].

At the molecular level, viral interactions between HBx from HBV and the delta antigen (HDAg) from HDV further enhance the oncogenic process [136].

HDV persistence occurs independently of the extent of the HBV replicative reservoir and is sustained by the continued production of HBsAg, which is primarily derived from integrated HBV DNA rather than from active HBV replication, contributing to an increased risk of HCC [137,138].

Consequently, HDV can persist even when HBV DNA levels are suppressed by antiviral therapy, as nucleoside analogs do not eliminate HBsAg expression from integrated sequences. This sustained production of HBsAg allows HDV virion assembly and continued infection. Furthermore, chronic HDV infection, combined with persistent immune-mediated liver damage and HBV-induced genomic instability, creates a pro-oncogenic environment that significantly increases the risk of HCC. These findings underscore the need for therapies that directly target HBsAg expression to achieve functional cure and reduce the risk of HCC in patients with HBV/HDV coinfection [135,139,140].

HDAg has been shown to interact with key cellular regulators, including RNA polymerase II, while simultaneously inducing oxidative stress, triggering DNA damage responses, and contributing to epigenetic dysregulation of host gene expression [141,142].

Furthermore, HDV may promote the clonal expansion of hepatocytes harboring HBV integrations, thereby contributing to the molecular evolution of hepatocarcinogenesis in chronic HBV-HDV coinfection.

### 2.7. The Role of HCV Coinfection in the Development of HCC

Hepatitis C virus (HCV) exerts an enhancing effect on HBV-associated hepatocarcinogenesis through a combination of virological interference, enhanced necroinflammatory activity, and convergent oncogenic signaling. In HBV/HCV coinfection, the two viruses establish a complex pattern of reciprocal suppression and episodic dominance, yet the net biological outcome is typically an intensification of hepatic damage and accelerated fibrogenesis [143]. HCV contributes additional carcinogenic pressure through the activity of core, NS3, and NS5A proteins, which modulate pathways such as NF-κB, JAK/STAT, PI3K/AKT, and TGF-β, promoting oxidative stress, epithelial–mesenchymal transition, and dysregulation of apoptosis [144,145]. These mechanisms act in parallel with HBV-specific drivers, resulting in cumulative genomic stress and a permissive environment for malignant transformation. Epidemiological data show that coinfected individuals exhibit a higher incidence of cirrhosis and hepatocellular carcinoma than patients with HBV monoinfection, even after adjustment for fibrosis stage [146]. Clinically, coinfection is characterized by more advanced portal hypertension, reduced hepatic reserve, and a higher likelihood of decompensation at HCC presentation, factors that negatively influence therapeutic eligibility and prognosis.

## 3. Current and Novel Anti-HBV Therapy: Role in Reducing HCC Development

### 3.1. The Impact of the Current Antiviral Therapy on HCC Risk

The currently approved treatment for chronic hepatitis B, based primarily on the usage of the nucleoside analogs (NUCs) entecavir and tenofovir, substantially reduces the risk of HCC development but fails to completely abrogate this risk.

In particular, a recent study has highlighted that among patients without advanced liver fibrosis, antiviral therapy can reduce HCC risk by 70%, but a cumulative 10-year HCC risk of 5% persists even with successful antiviral treatment [147].

This persistent oncogenic risk, present even in virologically suppressed patients, is related to the mechanisms of action of NUCs, which can efficiently suppress viral replication (in a rate of treated patients > 90%), but have very limited/no impact on early events occurring during the first phases of HBV replication, such as the formation of the cccDNA pool in the nuclei of hepatocytes and the integration of HBV DNA into the genome of the infected cells. In particular, the treatment with NUCs acts by inhibiting the reverse transcription of pregenomic HBV RNA, thus strongly reducing the formation of new relaxed circular DNA-containing virions, but does not directly affect the already established cccDNA reservoir. Indeed, there is evidence of cccDNA persistence even after long-term (>10 years) NUC treatment. Consequently, this intrahepatic reservoir can continue to produce viral proteins with oncogenic properties such as HBsAg and HBx, perpetuating the risk of neoplastic transformation of the hepatocytes. More recent findings analyzing liver biopsies from HBV chronically infected patients have highlighted that slow cccDNA decay under NUC treatment can occur even if at a much lower rate with respect to the decline of HBV DNA in serum under treatment [148]. In particular, it has been demonstrated that NUC treatment acts by reducing the number of hepatocytes positive to HBc protein, which likely represent cccDNA-containing cells, with a detrimental impact on cccDNA intrahepatic levels [149,150]. These findings have important implications in light of considering an earlier initiation of NUC treatment even in patients with a limited HBV replication and no/limited liver disease progression, currently not matching treatment criteria, since it could also have a positive impact on reducing the HBV intrahepatic reservoir, and in turn, can decrease the risk of progression toward HCC.

Moreover, it is relevant to remark that the high rate of virological suppression achieved by the current antiviral therapy, based on entecavir or tenofovir, results in a following reduction/abrogation of replication cycles in the nuclei of hepatocytes [46]. This also strongly limits the accumulation of genetic variability in the HBV genome and, thus, indirectly constrains the possibility for the selection of viral mutations, which can be associated with the acquisition of oncogenic properties.

Lastly, as previously described in this review, another mechanism that is currently recognized as a significant contributor to HBV-induced transformation of hepatocytes is represented by the integration of HBV DNA into the genome of infected hepatocytes.

Recently, it has been demonstrated that NUC therapy could also have an impact on HBV integration, thus also potentially affecting HCC risk related to HBV integration into critical regions of the hepatocytes’ genome. In particular, in a clinical trial including patients randomized to receive TDF or placebo for 3 years, it has been documented that TDF therapy led to a significant decrease in the number of transcriptionally active, distinct viral integrations compared to placebo [151]. However, more recent investigations showed that NUC treatment can prevent the formation of new integrations by reducing viral replication but has a little effect on pre-existing clonally expanded hepatocytes with integrated HBV DNA, and thus, on viral antigen expression from previously integrated HBV DNA [152].

Overall, the impact of antiviral therapy in reducing novel events of HBV DNA integration, as well as in preventing the accumulation of pro-oncogenic mutations, further reinforces the importance of also evaluating an earlier treatment initiation with NUC therapy in patients with a still limited disease.

**Figure 2:**
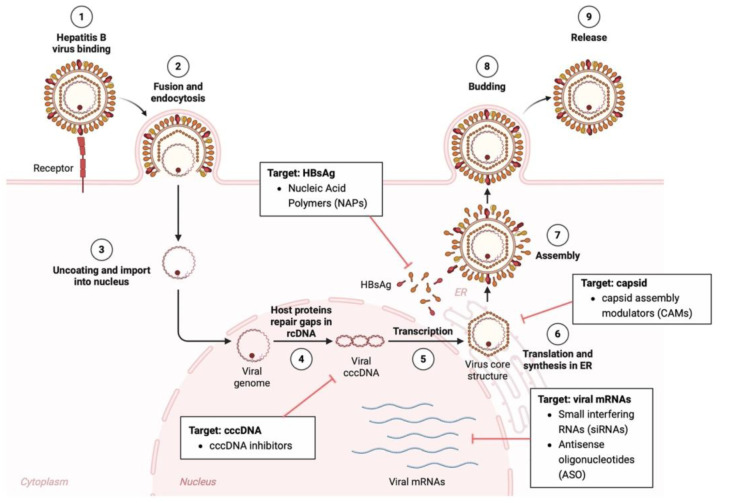
**Novel antiviral therapeutic strategies and their targets within the HBV life cycle.** This figure illustrates the intracellular replication cycle of HBV with a focus on current and investigational therapeutic strategies aimed at interfering with key steps of HBV replication cycle. Five major novel classes of antiviral drugs are highlighted: (i) nucleic acid polymers (NAPs) block the secretion of HBsAg, reducing its circulation and potentially restoring antiviral immune responses; (ii) cccDNA inhibitors aim to silence or eliminate this episomal DNA form in the nucleus, that is responsible for viral persistence and, in turn, its elimination is a critical step toward a functional HBV cure; (iii) capsid assembly modulators (CAMs) interfere with the correct assembly of the viral core, either by accelerating aberrant assembly or by destabilizing the capsid, thus preventing proper encapsidation of pregenomic RNA and viral polymerase; (iv) siRNAs and (v) antisense oligonucleotides (ASOs) are both compounds aimed to degrade or block the translation of viral mRNAs, leading to a reduction in viral protein expression and viral replication, supporting immune reactivation and antiviral control.

Otherwise, the persistent oncogenic risk, even under fully suppressive NUC therapy, calls for novel therapeutic strategies that could permit the achievement of HBsAg loss (rarely obtained by NUC therapy), an ideal therapeutic end-point that has been associated with a strong reduction in liver cancer [153,154]. To date, several novel anti-HBV therapies, based on antivirals (nucleic acid polymers, small interfering RNAs, Antisense oligonucleotides, cccDNA inhibitors, and capsid assembly modulators) and on immunomodulators (therapeutic vaccines, checkpoint inhibitors, and Toll-like receptor agonists) (Figure 2), are showing encouraging results in clinical trials, which will be discussed more extensively in the following paragraph. In light of this, these therapeutic agents could represent promising strategies for reducing the still high burden of HBV-related hepatocellular carcinoma [155,156,157,158].

### 3.2. Novel Therapeutic Strategies Against HBV Infection

Nucleic acid polymers (NAPs) are phosphorothioated oligonucleotides that inhibit the secretion of subviral particles of HBsAg, leading to a rapid and marked decrease in circulating HBsAg, promoting immune restoration, and increasing the chances of resolving chronic HBV infection (Figure 2) [159]. REP 2139, the most studied NAP, has shown potent antiviral effects in clinical trials, especially when combined with pegylated interferon or tenofovir, achieving long-term HBsAg clearance in some cases [160]. NAPs target HBsAg derived from both cccDNA and integrated HBV DNA, making them effective in all HBV genotypes [161]. Ongoing studies aim to confirm their safety and efficacy in larger populations. Overall, NAPs offer an innovative therapeutic approach by blocking HBsAg secretion rather than viral replication, potentially reducing the risk of HBV-related HCC.

Small interfering RNAs (siRNAs) are emerging as a promising therapeutic approach for chronic HBV infection, acting through RNA interference to degrade viral mRNAs and suppress the expression of HBV proteins, including HBsAg, core, polymerase, and HBX (Figure 2) [162,163]. Unlike nucleos(t)ide analogs, siRNAs target transcripts from both cccDNA and integrated HBV DNA, making them particularly effective at reducing persistent HBsAg levels. Several GalNAc-conjugated siRNA candidates, such as VIR-2218, JNJ-3989, and RG6346, are in clinical development and have demonstrated a significant reduction in HBsAg with good tolerability, although their long-term durability and efficacy in patients with integrated HBV DNA are still being studied [164,165,166,167]. In particular, VIR-2218, alone or in combination with other immunomodulators, has demonstrated high effectiveness in reducing HBsAg levels, and it is currently entering phase 3 clinical trials [167].

Antisense oligonucleotides (ASOs) are short, synthetic, single-stranded nucleic acid sequences designed to bind specifically to complementary HBV RNA transcripts [161,168].

ASOs targeting HBV include GSK3228836, also known as Beprovirsen, developed by GSK and Ionis Pharmaceuticals. Beprovirsen is a chemically modified ASO designed to reduce HBV RNA transcripts, thereby decreasing the production of HBsAg and other viral proteins (Figure 2). Early phase clinical trials have demonstrated significant reductions in HBsAg levels with a favorable safety profile. It is being evaluated both as monotherapy and in combination with nucleos(t)ide analogs [169,170]. Another ASO is RO7062931, developed by Roche, which is a GalNAc-conjugated ASO that targets HBV RNA to suppress viral gene expression [171]. Its hepatocyte-specific delivery enhances potency and minimizes off-target effects. Clinical trials are ongoing to assess its efficacy and safety. These ASOs exemplify RNA-targeting strategies that reduce viral antigen levels and complement existing therapies, aiming to achieve functional cure of chronic HBV infection.

Covalently closed circular DNA (cccDNA) is the stable nuclear reservoir of HBV and the main barrier to viral clearance, as it persists independently of HBV replication and is not affected by nucleos(t)ide analogs. Several strategies are currently being developed to edit, inhibit, or silence cccDNA (Figure 2). These include blocking its formation from relaxed circular DNA during viral entry and uncoating; modulating its transcription through epigenetic regulation with agents such as HDAC or bromodomain inhibitors; and targeting HBx, a viral protein essential for cccDNA activity, with compounds such as RG7834 [172,173].

Early clinical findings from the Phase 1 ELIMINATE-B study indicate that PBGENE-HBV (ARCUS), a nuclease-based in vivo gene-editing therapy delivered via lipid nanoparticles, can directly target the molecular reservoirs that sustain chronic HBV infection. In particular, this therapeutic approach is designed to cleave both episomal cccDNA and integrated HBV DNA, thereby reducing transcriptionally active viral targets, which are not addressed by current nucleos(t)ide analogs [174]. In the initial dose-escalation cohorts, PBGENE-HBV produced consistent, dose-dependent reductions in HBsAg, with on-target disruption of viral gene expression. The safety profile was favorable, with no dose-limiting toxicities or serious adverse events, supporting the feasibility of repeated dosing to enhance editing depth [175]. Mechanistically, the observed decline in HBsAg aligns with the predicted consequences of ARCUS-mediated cleavage of HBV DNA templates, which would reduce the production of subviral particles and potentially diminish the immunosuppressive burden imposed by high antigenemia [176]. Collectively, these early results position PBGENE-HBV as a promising candidate within the HBV cure pipeline, offering a distinct strategy aimed at depleting the stable viral reservoirs that underlie lifelong infection and persistent hepatocarcinogenic risk.

Capsid assembly modulators (CAMs) are direct-acting antivirals that target the HBV core protein, disrupting nucleocapsid assembly and inhibiting pgRNA packaging and reverse transcription (Figure 2) [177]. CAMs are classified as class I, which induce non-functional capsid aggregates, and class II, which accelerate premature capsid formation, preventing proper genome encapsulation [178,179]. In addition to suppressing viral replication, CAMs can also reduce cccDNA reconstitution, as nucleocapsids are involved in its formation [180]. Clinical candidates such as JNJ-6379, RO7049389, GLS4, and Vebicorvir (ABI-H0731) have shown significant reductions in HBV DNA and RNA, but only a limited reduction in HBsAg when used alone. CAMs are orally administered, well-tolerated, and are being studied in combination therapies to enhance antiviral efficacy [181,182,183,184].

Immunomodulators aim to restore antiviral immunity in chronic HBV by targeting immune exhaustion rather than the virus itself. Therapeutic vaccines (e.g., GS-4774, ABX203) enhance HBV-specific T cells; checkpoint inhibitors (e.g., nivolumab) reverse T cell exhaustion; Toll-like receptor (TLR) agonists (e.g., GS-9620 for TLR7, selgantolimod for TLR8) activate innate immunity and interferon pathways [185,186,187,188,189,190].

Pegylated interferon alpha, the only approved immunomodulator, can suppress HBV and induce a functional cure in some cases. Although mostly experimental, these agents, especially in combination with antivirals, can increase HBsAg loss and promote functional cure by overcoming immune tolerance [185].

Nitazoxanide (NTZ, Alinia), a drug approved in the United States by the FDA for the treatment of protozoan infections caused by *Cryptosporidium parvum* and *Giardia intestinalis* [191,192], has demonstrated broad-spectrum activity via antimicrobial activity in vitro against both Gram-positive and Gram-negative bacteria [193], as well as against a range of RNA and DNA viruses, including hepatitis B in cell culture assays [194,195,196,197,198,199,200,201]. NTZ potently blocks the association between HBx and the host factor DDB1. This disruption leads to a recovery of Smc5 protein expression and a marked reduction in viral RNA synthesis and antigen production, observed in both the HBV minicircle replication model and in primary human hepatocytes undergoing natural HBV infection [200]. A recent pilot clinical trial investigating the treatment of chronic HBV demonstrated that NTZ reduced serum HBV DNA levels and led to the clearance of HBsAg from the bloodstream [202].

Bulevirtide (BLV) is the first HBV/HDV entry inhibitor approved by the EMA with a high safety profile even in patients with advanced compensated cirrhosis. Suppression of HDV RNA induced by BLV alone or in combination with pegylated interferon α-2b (PegIFNα-2b) can improve liver function, reduce cirrhosis-related complications, and potentially slow the progression to HCC development [203].

## 4. Conclusions

The development of hepatocellular carcinoma represents the major complication of chronic HBV infection. To date, it is well recognized that HBV can induce carcinogenesis not only by indirect mechanisms of continuous immune-mediated liver damage and necro-inflammation but also by direct viral intrinsic factors. In particular, the occurrence of HBV DNA integration in genes modulating cell proliferation and the permanent cccDNA activity in all phases of HBV chronic infection, with the production of HBV pro-oncogenic proteins, underline the continuous tumorigenic risk, characterizing the entire course of HBV chronic infection. Of note, the development of HCC is remarkably reduced under NUC therapy; however, even under fully suppressive therapy with NUC, a residual HCC risk persists. Novel therapeutic strategies based on the use of both innovative direct antivirals and immune-therapeutics represent a potential weapon in the fight against the silent HBV pandemic, which affects 254 million people worldwide (3.3% of the global population) and causes over 1 million deaths per year [204]. To date, both approved and experimental therapies have shown promising results in achieving an HBV functional cure with HBsAg loss, a therapeutic end-point correlated with a stronger reduction in HCC risk. It is well known that specific mutations in HBsAg hinder the recognition of HBsAg by neutralizing antibodies, thus calling into question the effectiveness of HBV vaccination; therefore, the pharmacological approach to treating HBV infections represents an important challenge for the present and the future, which must go hand in hand with the development of new vaccination strategies [205].

In light of this, these novel therapeutic approaches could also be strongly beneficial for reducing the burden of HBV-related hepatocellular carcinoma. However, it should be carefully considered that only the final achievement of a sterilizing HBV cure, implying the complete elimination of cccDNA and integrated HBV DNA, could abrogate the risk of HCC development related to HBV chronic infection. So far, this represents an objective that is still unattainable, even with the new compounds under preclinical evaluation, and further drug development efforts, based on innovative approaches such as gene editing, should be explored to completely eliminate HBV infection and, thus, its related oncogenic progression.

## Data Availability

No new data were created.

## Abbreviations

The following abbreviations are used in this manuscript:

Akt	protein kinase B
AP-1	Activator Protein-1
ASO	antisense oligonucleotides
BLV	Bulevirtide
CAM	capsid assembly modulators
ccc DNA	covalently closed circular DNA
CCNA2	cyclin A2
CCND1	cyclin D1
CCNE1	cyclin E1
CD4	cluster of differentiation 4
CD8	cluster of differentiation 8
CDK	cyclin-dependent kinase
DDB1	Damage-Specific DNA Binding Protein 1
DSL	double-stranded linear
eDNA	extrachromosomal circular DNA
ER	endoplasmic reticulum
ERK	extracellular signal-regulated kinases
Forkhead Box O-1	FOXO1
GLI2	GLI family zinc finger 2
HBc	HBV core protein/antigen
HBeAg	HBV secreted protein/antigene
HBsAg	HBV surface glycoprotein/antigen
HBV	hepatitis B virus
HBx	HBV regulatory X protein
HCC	hepatocellular carcinoma
HCV	hepatitis C virus
HDAg	delta antigen
IL-1	interleukin-1
IL-5	interleukin-5
IL-6	interleukin-6
KMT2B	lysine methyltransferase 2B
L-HBsAg	large HBV surface glycoprotein
MAPK	mitogen-activated protein kinase
MEK	MAPK/ERK Kinase
MHC	Major histocompatibility complex
M-HBsAg	medium HBV surface glycoprotein
MLL4	Mixed lineage leukemia-4
NAFLD	non-alcoholic fatty liver disease
NAP	nucleic acid polymer
NF-κB	nuclear factor-kappa B
NHEJ	non-homologous end joining
NTZ	nitazoxanide
NUCs	Nucleos(t)ide analogs
ORF	open ready frame
p53	tumor suppressor protein 53
PAI-1	Plasminogen Activator Inhibitor-1
PegIFNα	pegylated interferon α-2b
pg RNA	pregenomic RNA
pRB	retinoblastoma tumor suppressor protein
rcDNA	relaxed circular DNA
S-HBsAg	small HBV surface glycoprotein
siRNA	small interfering RNA
Smc	Structural Maintenance of Chromosomes
TERT	Telomerase reverse transcriptase
TLR	Toll-like receptor
WT	wild-type
